# The soft computing-based approach to investigate allergic diseases: a systematic review

**DOI:** 10.1186/s12948-017-0066-3

**Published:** 2017-04-13

**Authors:** Gennaro Tartarisco, Alessandro Tonacci, Paola Lucia Minciullo, Lucia Billeci, Giovanni Pioggia, Cristoforo Incorvaia, Sebastiano Gangemi

**Affiliations:** 1 Messina Unit, National Research Council of Italy (CNR)-Institute of Applied Science and Intelligent System (ISASI), Messina, Italy; 2 Pisa Unit, National Research Council of Italy (CNR)-Institute of Clinical Physiology (IFC), Pisa, Italy; 3 School and Division of Allergy and Clinical Immunology, Department of Clinical and Experimental Medicine, University Hospital “G. Martino”, Messina, Italy; 4 Cardiac/Pulmonary Rehabilitation, ASST PINI/CTO, Via Bignami 1, Milan, Italy

**Keywords:** Allergy, Artificial intelligence, Artificial neural networks, Asthma, Fuzzy logic

## Abstract

**Background:**

Early recognition of inflammatory markers and their relation to asthma, adverse drug reactions, allergic rhinitis, atopic dermatitis and other allergic diseases is an important goal in allergy. The vast majority of studies in the literature are based on classic statistical methods; however, developments in computational techniques such as soft computing-based approaches hold new promise in this field.

**Objective:**

The aim of this manuscript is to systematically review the main soft computing-based techniques such as artificial neural networks, support vector machines, bayesian networks and fuzzy logic to investigate their performances in the field of allergic diseases.

**Methods:**

The review was conducted following PRISMA guidelines and the protocol was registered within PROSPERO database (CRD42016038894). The research was performed on PubMed and ScienceDirect, covering the period starting from September 1, 1990 through April 19, 2016.

**Results:**

The review included 27 studies related to allergic diseases and soft computing performances. We observed promising results with an overall accuracy of 86.5%, mainly focused on asthmatic disease. The review reveals that soft computing-based approaches are suitable for big data analysis and can be very powerful, especially when dealing with uncertainty and poorly characterized parameters. Furthermore, they can provide valuable support in case of lack of data and entangled cause–effect relationships, which make it difficult to assess the evolution of disease.

**Conclusions:**

Although most works deal with asthma, we believe the soft computing approach could be a real breakthrough and foster new insights into other allergic diseases as well.

## Background

Recent advances in healthcare innovation have challenged us to think about the pioneering potential of big data coming from the digital world to invade the medical field. Big data introduces the exciting technological ability to digitize human beings in order to achieve a real personalization of medicine. Soft computing (SC) methods possess the extraordinary ability to exploit meaningful relationships of digital big data, making them suitable for the diagnosis, treatment and prediction of the outcome in many clinical scenarios. In the field of allergy these methods may be extremely useful to obtain important data and information on the characteristics and the management of many allergic diseases. Existing literature on the relationship between SC models and allergic diseases will be presented and discussed in this paper, highlighting the novel perspectives of this pioneering approach.

## Soft computing methods

SC is a branch of computer science introduced in the early 1990s [1]. It includes a collection of techniques that resemble biological processes more closely than traditional methodologies. SC deals with approximate reasoning, imprecision and uncertainty in order to achieve robustness and low-cost solutions for complex data analysis. This approach could excel in modern medicine, where the analysis and application of a large amount of knowledge are necessary to solve complex clinical problems, which in most cases are not deterministic. Table 1 lists the most important fields of application of SC methodologies in medicine.

**Table 1:** Main soft computing uses in medicine

Main applications
Classification and prediction of disease categories
Diagnosis and prognosis
Medical decision-making processes
Physiological signal analysis
Epidemiological studies
Genetic association studies
Pharmacokinetics
Imaging
Geo-spatial distribution of diseases

**Fig. 1:**
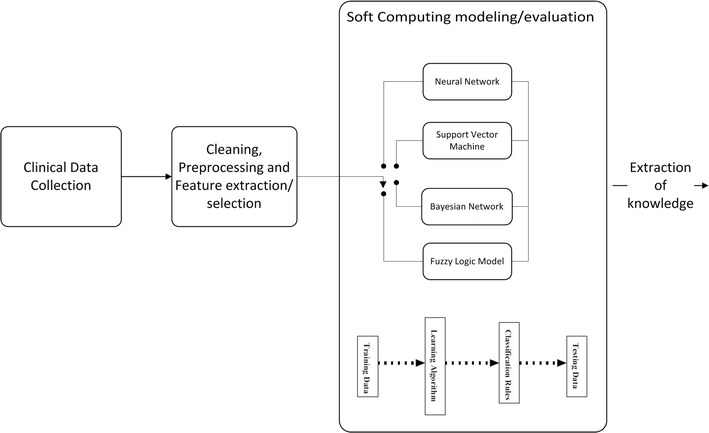
Overview of soft computing-based data analysis process

SC models encompass automatic computing procedures, without human intervention, and are able to learn a task from a series of training examples. Moreover, they aim to generate sufficient output simple enough to be easily understood by the humans. Differently, classic statistical approaches are generally characterized by having an explicit model of probability, with the assumption that in most cases they require the intervention of an expert with regard to variable selection, transformation and overall structuring of the problem [2]. The general approach of SC modeling data analysis typically consists of four stages as shown in Fig. 1: (i) collection and encoding of clinical data in an electronic form suitable for further processing; (ii) data processing with techniques of feature extraction and dimension reduction (i.e., principal component analysis), selecting the most predictive parameters; (iii) pattern modeling selecting an SC model; (iv) extraction of knowledge by evaluating accuracy, sensitivity and specificity.

In the third step of Fig. 1, the most common SC models considered in this systematic review are shown: artificial neural networks (ANN), support vector machines (SVM), bayesian networks (BN), and fuzzy logic (FL).

### Artificial Neural Networks

**Fig. 2:**
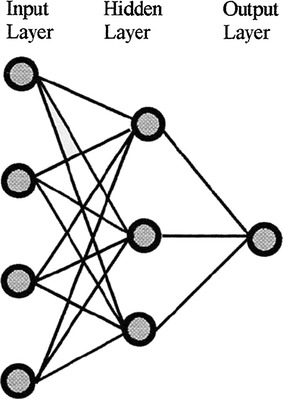
The topology of multi-layer perceptron neural network to screen a population according to individual likelihood of asthma [4]. It is composed by interconnected nodes structured in three main layers. The input nodes represents questionnaire responses and the single output node represents probability of asthma

The ANN is a flexible non-linear model inspired by the brain’s interconnections. ANNs possess an adaptable knowledge that is distributed over many neurons and synaptic connections. They are generally based on interconnected nodes (neurons), processing units able to compute input, activation and output functions. Each connection (synapse strength) is provided by a weight adapted during the learning phase. The most common example of ANN is the multi-layer perceptron (MLP) [3]. The topology of the network is composed by interconnected nodes arranged in multiple layers. In the first layer each node corresponds to each input variable. The layers in the middle (hidden layers) represent the core of the non-linear model while the number of hidden nodes represents the complexity of the network. The relationships among variables are built using a sufficient number of training data and represented as functions using methods such as maximum likelihood estimation, maximum a posteriori or back propagation. The utility of ANN models lies in the fact that they can be used to infer a function from observations (training data). This is particularly useful in applications where the complexity of the data or tasks makes designing such a function by hand impractical. An interesting example of MLP, as shown in Fig. 2, was proposed by Hirsch et al. in 2001 to analyze an enormous amount of surveys to screen a population for asthma [4]. The trained neural network received as input 6825 screening questionnaires and was able to predict a final diagnosis of asthma with an accuracy of 74%.

### The support vector machine

The SVM [5] is one of the most common machine learning models able to map the N input variables with a kernel function in an N-dimensional space (N-hyperplanes). The model is based on an algorithm able to find the best separating hyperplanes (maximum-margin hyperplane). Typically, SVM models are used for classification and regression analysis.

### The Bayesian networks

**Fig. 3:**
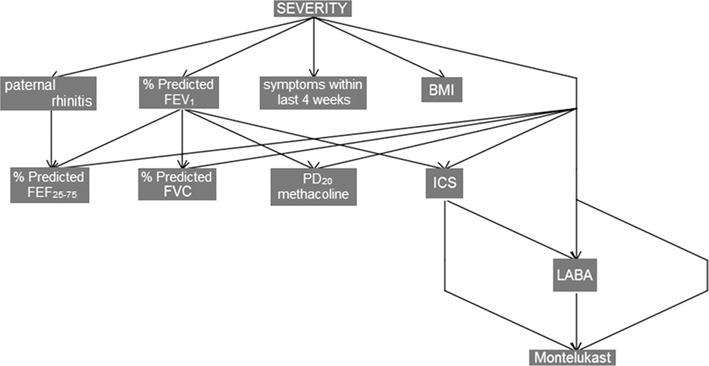
Bayesian networks model evidences conditional dependencies between severity as diagnosed by the physician and the variable space selected by a stepwise search [6]. *BMI* body mass index, *FEF* forced expiratory flow, *ICS* inhaled corti-costeroids, *LABA* long-acting β_2_-agonists

The BN are suitable for providing a graphical representation of variables and their complex relationships. BN have the advantage of creating predictive models directly from data. The topology is an acyclic graph in which a set of nodes represents the variables, while the edges between nodes represent the probabilistic relationships between variables. More specifically, a node with an incoming arrow is conditioned by the node from which the arrow originates. Despite traditional regression approaches, BN are more flexible and accurate in small samples if we incorporate correct prior information and advantageous in handling missing data, which is prevalent in the clinical field. Moreover, they are not limited to representing the dependencies of a single outcome variable on predictor variables. Figure 3 reports an example of a BN model for studying asthma severity of Prosperi et al. [6]. The model reported in a tree topology was able to explain the main dependencies between severity and variables such as body mass index (BMI), forced expiratory flow (FEF), inhaled corti-costeroids (ICS), long-acting β_2_-agonists (LABA) after a stepwise search in the whole original variable space.

### The fuzzy logic model

**Fig. 4:**
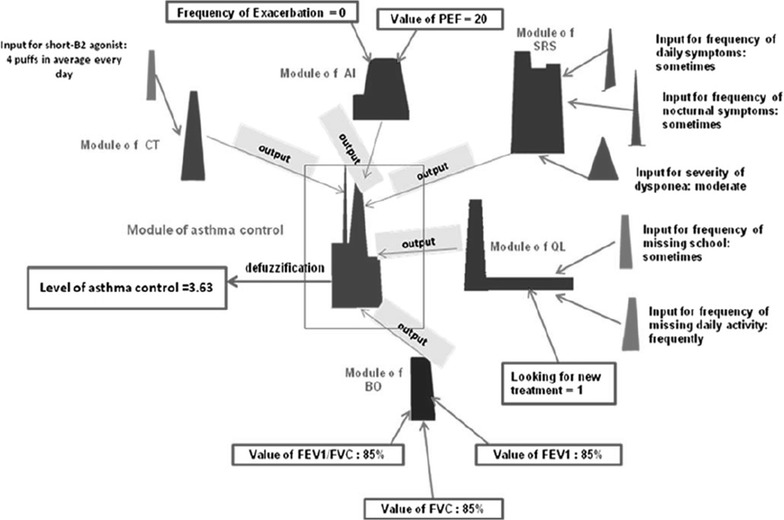
Schematic view of fuzzy logic model able to combine input variables related to severity of respiratory symptoms (SRS), quality of life (QL), current medical treatment (CT), instability of asthma (AI), bronchial obstruction (BO) to infer the level of asthma control (AC) [8]

While ANN, SVM and BN are important examples of SC models based on mathematical structures underlying learning, the FL approach [7] is based on integration of structured human knowledge into workable algorithms. Input and output of FL model are defined, converted to linguistic parameters (fuzzification) and the relationship among variables is generated through a set of rules (inference rules) defined by the experts. Finally, the output is represented by the aggregation of obtained results of input modules, converted into a numerical value (defuzzification) and classified. The FL approach is an alternative to the classic statistical methods where every proposition must either be “true” or “false”. Instead, fuzzy logic asserts that things can be simultaneously “true” and “not true”, with a certain membership degree to each class. FL techniques are used to deal with uncertainty and can be very powerful when there are poorly characterized parameters. In Fig. 4 an example of FL model provided by Zolnoori et al. to predict the level of asthma controls is reported [8]. The system is composed of 14 variables organized in five modules related to respiratory symptoms severity (SRS), bronchial obstruction (BO), asthma instability (AI), current treatment (CT), and quality of life (QL). All these variables are represented with fuzzy rules defined by experts and then aggregated in a fuzzy network. The output of the system is given by the degree of asthma control classified in five categories: excellent (0–1), good (1–3), fair (3–5), poor (5–7), and very poor (7–10).

## Methods

### Literature search

The research was performed on PubMed and ScienceDirect, covering the period starting from September 1, 1990 through April 19, 2016. We explored studies dealing with the most frequently adopted SC models (ANN, SVM, BN, FL) and allergic diseases. Research in PubMed was performed using medical subject headings (MeSH^®^) to report the most common SC methodologies employed to study the most frequent allergic diseases included under the Mesh term “hypersensitivity”. The keywords used to search were based on the following logical linguistic pattern: (“Hypersensitivity”[Mesh]) AND (“Neural Networks [Computer]”[Mesh]) OR (“Support Vector Machines”[Mesh]) OR (“Bayes Theorem”[Mesh]) OR (“Fuzzy Logic”[Mesh]). Instead, the electronic search strategy on ScienceDirect was performed with the following queries: (“asthma” or “adverse drug reactions” or “allergic rhinitis” or “atopic dermatitis” or “allergic conjunctivitis”) and (“artificial neural networks” or “support vector machine” or “Bayesian network” or “fuzzy logic”).

### Inclusion and exclusion criteria

The research was limited to clinical cross-sectional studies and case–control studies of articles published in peer-reviewed journals. Case–study reports, genetic association studies, cost-effectiveness healthcare studies, pollen/climate changes and classification of respiratory sounds were discarded from the review.

### Study selection

The research was conducted independently by two authors, who evaluated whether the information of each reference was relevant or not. Each disagreement between the two reviewers was resolved by discussion until a consensus was reached. If the abstract did not include enough information to evaluate inclusion or exclusion, the full text of publication was reviewed if available. Otherwise, the paper was excluded. The selected papers were sorted by relevance and grouped for each allergic disease (Table 3). In this report, we first review recent findings for SC model-related allergic diseases (summarized in Table 2), evaluating the accuracy, sensitivity and specificity of SC models. We then critically discuss the potential strength and future implications for research in this field.

**Table 2:** Studies dealing with SC models and allergic diseases

Authors	Application	Subjects	Description	Input features	SC model	Findings
Prosperi et al. [6]	To predict asthma severity	383 children with asthma (age 6–18 years)	Use of unsupervised statistical learning techniques, such as exploratory factor analysis (EFA), hierarchical clustering (HC) to identify asthma phenotypes	Lung function, inflammatory and allergy markers, family history, environmental exposures, body mass index, age of asthma onset and medications	BN	Significant recognition of asthma severity
Farion et al. [12]	To predict asthma exacerbation	322 Phase 1: 240 children (age 1–17 years) Phase 2: 82 children (age 1–17 years)	Phase 1: selection of the most accurate machine learning model with WEKA tool Phase 2: comparison of performance of BN with PRAM score and Physicians	42 attributes corresponding to the patient’s history, current asthma exacerbation, primary assessment and a selected secondary assessment	BN	Phase 1: Accuracy = 68% Phase 2: Accuracy = 70.7%
Finkelstein et al. [13]	To predict asthma exacerbation	26 adult asthmatic patients	Use of a modern tele-monitoring system at home	Daily self-reports	SVM	Accuracy = 80% Sensitivity = 84% Specificity = 80%
Sanders et al. [10]	To predict asthma exacerbation	4023 patients (age 2–18 years)	Use of a SC model to identify patients eligible for asthma care guidelines	Past diagnoses, allergies, family history, medications, social history and vital signs (temperature, respiratory rate, and oxygen saturation)	BN	Accuracy = 96% Sensitivity = 90% Specificity = 88.3%
Dexheimer et al. [11]	Same of Sanders et al. [10]	4023 patients (age 2–18 years)	Comparison of machine learning models to best identify patients eligible for an asthma care guideline	Same of Sanders et al. [10]	ANN,BN, Gaussian processes (GP)	BN accuracy = 96% GP accuracy = 95.6% ANN accuracy = 94%
Pifferi et al. [20]	To classify asthma control levels	77 patients (age 7.5–17 years)	Assessment of spirometry and fractional exhaled nitric oxide (FeNO) measurements to classify asthma control according to GINA guidelines	1st model: values of spirometry; 2nd model: values of FeNO; 3rd model: values of spirometry and FeNO	ANN	3rd model achieved best performances of classification Accuracy = 86.4%
Pifferi et al. [17]	To classify asthmatic vs control	123 90 asthmatic children (age 9–16 years) and 33 controls (age 12–13 years),	Pattern recognition analysis of the exhaled breath temperature curve	The rate of temperature increase and the mean plateau value	ANN	Accuracy = 5% Sensitivity = 77.2% Specificity = 99%
Jaing et al. [21]	To classify how children manage their asthma	305 children (age 5–14 years)	Each participant was given 10 asthma-based problems and asked to manage them	Each management decision and its order	ANN	Significant classification of five major classes representing different approaches to solving an acute asthma case
Kharroubi et al. [24]	To classify health state of patient with asthma	307 subjects	Estimation of a preference-based index for asthma (five-dimensional asthma quality of life utility index)	99 features about health statuses	BN	BN model is more appropriate than conventionally used parametric random-effects model
Hirsch et al. [4]	To classify asthmatic vs control	6825 adults (age ≥ 16)	Respiratory questionnaires were analyzed by experts and compared with results provided by neural network	12 answers provided by the respiratory questionnaire (wheezing, chest tightness, shortness of breath, night cough), family history of asthma and associated conditions of hay fever or eczema	ANN	Accuracy = 74%
Chatzimichail et al. [19]	To classify asthmatic vs control	112 children (age 7–14 years)	Three step analysis:1-feature selection with Principal Component Analysis, 2-pattern classification, 3-performance evaluation	46 prognostic factors including data on asthma, allergic diseases, and lifestyle factors	SVM	Accuracy = 95.54% Sensitivity = 95.45% Specificity = 95.59%
Goulart et al. [32]	To classify allergic conjunctivitis vs control	102 48 with allergic conjunctivitis and 54 controls (age 3–14 years)	Allergic conjunctivitis questionnaires were analyzed by experts and compared with results provided by neural network	7 items selected from a questionnaire of 15 answers	ANN	Accuracy = 100%
Takahashi et al. [33]	To classify atopic dermatitis vs control	4610 answers, 2714 infants (12 months old) and 1896 children (2 years old)	To analyze the predictive accuracy of the predictive model for effect of atopic dermatitis in infancy, from the data of the epidemiological survey	Family history (father, mother, siblings, grand-father, grand-mother), food restriction, food allergy, age, food restriction of mother, egg introduced time, cow’s milk introduced time	ANN	Accuracy = 96.4% Sensitivity = 88.6% Specificity = 99.5%
Christopher et al. [31]	To classify allergic rhinitis vs control	872 patients of all age groups	Allergic rhinitis reports of intradermal skin tests were analyzed by experts and compared with results provided by neural network	Patient’s history and 40 clinically relevant allergens	ANN	Accuracy = 88.31% Sensitivity = 88.3% Specificity = 88.2%
De Matas et al. [23]	To predict the clinical effect of salbutamol	23 12 healthy volunteers and 11 mild asthmatics	In vivo and in vitro data of human subjects were analyzed using SC modeling	Demographic data and urinary levels of salbutamol and metabolite	ANN	Accuracy = 83.5%
De Matas et al. [22]	To predict the clinical effect of salbutamol	18 mild-moderate asthmatic patients	SC modeling to predict the bronchodilator response at 10 (T10) and 20 (T20) min after receiving each of the 4 doses for each of the 3 different particle sizes	Aerodynamic particle size (APS), body surface area (BSA), age, pre-treatment forced expiratory volume in one-second (FEV1), forced vital capacity, cumulative emitted drug dose and bronchodilator reversibility	ANN	Accuracy = 88%
Gandhi et al. [25]	To predict hypersensitivity reaction	2458 reports concerning thrombotic events selected from AERS (adverse event reporting system) database	Retrospective analysis focused on thrombotic events associated with C1 esterase inhibitor products	Adverse events; demographic and administrative information; drug/biologic information; report sources; patient outcomes; drug therapy start and end dates; indications for use/diagnosis	BN	Potential signals of C1 esterase inhibitor product—associated thrombotic events among patients with hereditary angioedema were identified
Naranjo et al. [28]	To predict the posterior probability of a drug (BARDI tool)	51 patients	BARDI tool, calculates the posterior probability of a drug being the cause based on epidemiologic and case data	Reactions after receiving aromatic anticonvulsants	BN	Accuracy = 93% Sensitivity = 94% Specificity = 50%
Lanctot et al. [29]	To predict the posterior probability of a drug (BARDI tool)	27 cases of skin reactions	BARDI, combined with the LTA, a biochemical test that determines the percent of cell death because of toxic metabolites of a drug	Skin reactions associated with sulfonamide therapy	BN	Accuracy = 96% Sensitivity = 79% Specificity = 38%
Lanctot et al. [30]	To predict the posterior probability of a drug (BARDI tool)	106 challenging cases	BARDI, compared with the Adverse Drug Reaction Probability Scale (APS)	Drug- and nondrug-induced adverse events	BN	BN model discriminate better than ADR drug from nondrug-induced cases.
Kadoyama et al. [26]	To predict hypersensitivity reaction caused by anticancer agents	1,644,220 reported cases from 2004 to 2009 (AERS database)	SC model to detect important pattern related to anticancer agents-associated adverse events	Adverse events; demographic and administrative information; drug/biologic information; report sources; patient outcomes; drug therapy start and end dates; indications for use/diagnosis	BN	Potential signals were detected for paclitaxel-associated mild, severe, and lethal hypersensitivity reactions, and docetaxel-associated lethal reactions
Sakaeda et al. [27]	To predict hypersensitivity reactions caused by platinum agents	1,644,220 reported cases from 2004 to 2009 (AERS database)	The BN analysis aims to search for previously unknown patterns and automatically detect important signals, i.e., platinum agent-associat d adverse events, from such a large database	Adverse events; demographic and administrative information; drug/biologic information; report sources; patient outcomes; drug therapy start and end dates	BN	Significant association between the platinum agent-and mild, severe, and lethal hypersensitivity reactions
Lurie et al. [16]	To classify asthma severity	113 patients (age 42.9 ± 16.3 years)	Implementation of a fuzzy model able to combine patients’ and doctors’ asthma perceptions	Doctor assessment, variables self-assessed by patients (dyspnea, perceived treatment efficacy, asthma-related quality of life questionnaire (AQLQ)), patients’ sociodemographic characteristics, and asthma characteristics	FL	Accuracy = 73%
Zolnoori et al. [8]	To classify asthma control level	42 asthmatic patients	Implementation of a fuzzy model able to estimate the level of asthma control and help physicians to manage their patients more effectively	Respiratory symptom severity, bronchial obstruction, asthma instability, current treatment and quality of life	FL	Accuracy = 100%
Zolnoori et al. [14]	To classify asthma exacerbation	25 patients	Implementation of a fuzzy model able to estimate the level of asthma exacerbation and help physicians to manage their patients more effectively	Status of breathless, status of wheeze, status of alertness, status of respiratory rate, status of talk, status of pulse/min heart rate, value of PEF after initial bronchodilator, value of paCO_2_, value of SaO_2_%	FL	Accuracy = 100%
Zolnoori et al. [15]	To classify asthma severity	28 patients	Implementation of a fuzzy model able to estimate the four categories of asthma severity and help physicians to manage their patients more effectively	Bronchial obstruction, response to drug, skin prick test, severity of respiratory symptoms, instability of asthma, IgE value, quality of life	FL	Accuracy = 100%
Zolnoori et al. [18]	To classify asthmatic vs control	278 139 asthmatic patients and 139 non-asthmatic patients (age range 6–18)	Implementation of a fuzzy model to help physicians to manage their patients more effectively	Medical history, environmental factors, allergic rhinitis, genetic factors, consequences of asthma on lung tissues, response to laboratory tests and response to short-term medicine	FL	Sensitivity = 88% Specificity = 100%

*SC* soft computing, *ANN* artificial neural networks, *SVM* support vector machines, *BN* Bayesian networks, *FL* Fuzzy logic, *PRAM* pediatric respiratory assessment measure. *GINA* global initiative for asthma

## Results

**Fig. 5:**
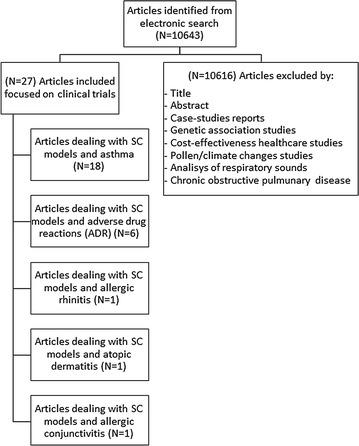
Search strategy used to select articles included into this review

We identified 10,643 references from citation database queries, respectively 10,486 from ScienceDirect and 157 from PubMed. The systematic review, whose details are shown in Fig. 5, revealed 27 papers dealing with clinical trials related to allergic diseases and SC models in the above-mentioned period.

In the present systematic review, the selected papers were grouped according to the specific type of allergy (Table 3): 18 works on asthma detection and diagnosis, six on adverse drug reactions (ADR), one on allergic rhinitis, one on allergic conjunctivitis and one on atopic dermatitis.

**Table 3:** Overview of clinical studies related to SC models and allergic diseases

Type of allergy	No. of studies	Overall accuracy (%)	Application	ANN	SVM	BN	FL
Asthma	18	82.44 ± 23.71	To classify exacerbations [10–14]	1	1	2	1
			To classify severity [6, 15, 16]	–	–	1	2
			To classify pathologic vs control [4, 17–19]	2	1	–	1
			To classify asthma control level [8, 20]	1	–	–	1
			To classify how manage their pathology [21]	1	–	–	–
			To predict the clinical effect of salbutamol [22, 23]	2	–	–	–
			To classify health state[24]	–	–	1	–
ADR	6	94.5 ± 2.12	To predict the posterior probability of a drug (BARDI tool) [28–30]	–	–	3	–
			To predict hypersensitivity reaction (AERS database) [25–27]	–	–	3	–
Allergic rhinitis	1	88.31	To classify pathologic vs control [31]	1	–	–	–
Allergic conjunctivitis	1	100	To classify pathologic vs control [32]	1	–	–	–
Atopic dermatitis	1	96.4	To classify pathologic vs control [33]	1	–	–	–

### Studies on asthma

Findings from clinical studies about asthma suggest that SC models are mainly suitable for classification of exacerbations, severity, recognition of asthmatic patients vs. controls and for asthma level control. Predicting the correct category of exacerbation severity is challenging in order to assess the appropriate hospitalization of the patient [9]. Sanders et al. [10] first proposed an SC model to detect asthma exacerbation in patients from the pediatric emergency department. They employed a BN model analyzing variables related to past diagnoses, allergies, family history, medications, social history and vital signs (temperature, respiratory rate, and oxygen saturation). The output of the network was the probability of a patient being admitted to the emergency department with asthma exacerbation and being eligible for treatment using asthma-care guidelines (GINA). The implemented model was able to identify guideline-eligible patients with an accuracy of 96%. Dexheimer et al. [11], using the same database as Sanders et al. [10], compared BN and other SC methodologies such as ANN and Gaussian processes, for identifying asthma exacerbations. Here, the accuracies achieved were 96, 95.6 and 94%, respectively, with no significant differences. In a recent prospective study, Farion et al. [12] compared different SC models using tenfold cross-validation and BN achieved the best performances. In a second phase of work they compared BN results with predictions derived by the pediatric respiratory assessment measure (PRAM) score [10] and with those made by physicians, obtaining high comparable results. In another study, Finkelstein et al. [13] integrated the SC methodologies in a decision support home telemonitoring platform to predict asthma exacerbations. The study dataset was based on daily self-reports administered on 26 adult asthmatic patients at home. All the collected data were analyzed with a BN classifier and an SVM able to predict asthma exacerbation with an accuracy of 80%. Zolnoori et al. [14] developed an intelligent clinical decision support system (CDSS) based on the FL model to assess the level of asthma exacerbation. Input variables included the status of breathlessness, status of wheeze, status of alertness, status of respiratory rate, status of talk, status of pulse/min heart rate, value of PEF after initial bronchodilator, value of paCO_2_, value of SaO_2_%. The model was able to classify patients in four categories of asthma exacerbation including mild, moderate, severe and respiratory arrest imminent (RAI), achieving an accuracy of 100% (Cohen’s coefficient k = 1). The studies analyzed so far suggested the high accuracy of an SC method to detect the correct level of asthma exacerbation. Within this review we also examined the role of SC methodologies in classifying the severity of asthma. Zolnoori et al. [15], in a second study, evaluated asthma severity by implementing a fuzzy rule expert system composed by seven input modules. Analyzed variables included bronchial obstruction, response to drugs, skin prick test, severity of respiratory symptoms, instability of asthma, IgE antibodies value and quality of life. This work evidenced a complete correspondence between model’s and physician’s evaluation (mild intermittent, mild persistent, moderate persistent, severe persistent) with and accuracy of 100% (Cohen’s coefficient k = 1). In another study, Laure et al. [16] proposed the fuzzy approach to model the patient’s perception of asthma severity. The model included variables self-assessed by patients (dyspnea, perceived treatment efficacy, asthma-related quality of life questionnaire (AQLQ), patients’ socio-demographic characteristics, and asthma characteristics. The output of the model was compared with doctor assessment of asthma severity according to (GINA) guidelines. The study highlighted a clear tendency of the patient to underestimate asthma severity compared to the doctor assessment. This finding suggest that assessment of asthma severity should consider both patients’ and doctors’ perceptions of the disease and should include an AQLQ measure. Prosperi et al. [6] adopted BN to analyze non-linear relationships among variables and identified prognostic factors of asthma severity. Input variables included lung function, inflammatory and allergy markers, family history, environmental exposures, body mass index, age of asthma onset and medications. SC methodologies were found to help investigators to identify complex patterns and structures in the data, despite needing a thoughtful selection of input features and an appropriate data labeling in the case of identification of real asthma subgroups. Other studies examined the capabilities of SC methodologies to distinguish asthmatic patients from controls. Pifferi et al. [17] tested an ANN by extracting input features from the exhaled breath temperature curve (i.e., the rate of temperature increase and the mean plateau value). The model was tested in 90 asthmatic children and 33 healthy age-matched controls. ANN was able to recognize asthmatic children and controls with an accuracy of 99.3 and 70.3% respectively. In another study, an FL model was developed, and a sensitivity of 88% and specificity of 100% was obtained for a cutoff value of 0.7 of ROC curve [18].

These studies show the utility of an intelligent patient-based SC system to support asthma diagnosis, especially in developing countries, because of limitations on access to medical specialists and laboratory facilities. This review revealed that the use of SC methodologies could also have an important impact on analyzing huge amounts of screening questionnaires and to predict allergic diseases. In 2001 Hirsch et al. [4], proposed the use of ANN to screen a population for asthma, using the responses to a respiratory questionnaire. A random sample of 180 from 6825 respondents to a community survey underwent clinical review. Each survey was labeled according to likelihood of asthma, combining three independent expert opinions. The ANN was trained using the 12 questionnaire responses as input and the probability labels as outputs. Using the known probability labels from the training set, it was possible to derive the expected proportion of true asthmatic patients. In 2013 Chatzimichail et al. [19] proposed an intelligent approach based on SVM for asthma prediction in symptomatic preschool children based on questionnaire analysis. In this study 112 patients ranging from 7 to 14 years of age were analyzed. The performances of the SVM were evaluated by using the tenfold cross-validation approach and achieved an accuracy of 95.54%. Some studies emphasized the use of SC methodologies to objectivize the categorization of asthma control levels. Pifferi et al. [20] developed three ANNs (multi-layer perceptron) able to classify children with allergies according to three classes provided by GINA assessment (controlled, partially controlled and uncontrolled asthma) using only the input value of two simple measurements, namely spirometry and fractional exhaled nitric oxide (FeNO). Among the three tested models, only the input combination of values of spirometry and FeNO was able to provide high accuracy. More specifically, the model was able to recognize 100% of children with uncontrolled asthma, 74% with partially controlled asthma and 99% with totally controlled asthma. The same work presented a cross-sectional study of 77 children with allergic asthma. In this case the selected ANN model prospectively identified correctly 100% of uncontrolled, 79.5% of partially controlled and 79.6% of the controlled children. In another work, based on an FL model to predict the asthma control level, the clinical features of spirometry were combined with another four input classes of variables provided by the patients: respiratory symptom severity, asthma instability, current treatment and quality of life. The model was able to discriminate five categories of asthma control level: very poor, poor, fair, good, and excellent, achieving an accuracy of 100% (Cohen’s coefficient k = 1) [8]. These findings suggest that the combination of clinical features (i.e., spirometry) and the subjective information provided by asthmatic patients can improve the performance of classification of SC models and help physicians to manage their patients more effectively. Another study demonstrates how SC models, through a training and pattern-recognition approach, can solve classification problems even when the specific category is not well-defined. In this regard, in a pilot study Jaing et al. [21] proposed the use of ANN to identify children’s behavior categories representing different approaches of asthma management. This review also revealed that SC models are potentially useful for estimating pharmacokinetics performances. In two clinical trials, De Matas et al. [22, 23] used the ANN to model in vitro and in vivo data to predict the clinical effects of dry powder inhaler formulations containing salbutamol sulfate in individual subjects. In the first study, on healthy subjects the trained ANN was able to predict 83% of cumulative urinary excretion of salbutamol and metabolite 0–24 h post-inhalation. The correct prediction of ANN for mild asthmatic patients was 84%. In the second study, the ANN model was used to forecast the bronchodilator response defined as ΔFEV1 (%) measured at both 10 (T10) and 20 (T20) minutes after receiving each of the four doses of salbutamol sulfate puffs for each of the three different particle sizes (1.5, 3 and 6 µm).The average error between predicted and observed ΔFEV1(%) for individual subjects was <4% across the cumulative dosing regimen. These findings provide further evidence that ANNs supply a suitable approach for modeling complex biological data sets and have the potential to generate predictable models that can provide reliable estimations of clinical response to inhaled drug products in humans. The last clinical study about SC models and asthma disease was proposed by Kharroubi et al. [24], who explored the use of a non-parametric Bayesian method to classify the health state of people with asthma. The work presents the results of the non-parametric model compared to the original model estimated using a conventional parametric random-effects model. This work evidenced that the non-parametric Bayesian models are theoretically more appropriate than previously used parametric models and provide better estimates of asthma quality of life.

### Studies on adverse drug reactions

This review examined results about the use of SC models and adverse drug reactions (ADRs). Three studies evaluated the ADRs from the public version of the food and drug administration (FDA) adverse event reporting system (AERS). A large dataset of 1,644,220 case reports from 2004 to 2009 was analyzed through the use of a BN. In particular, Gandhi et al. [25] identified 10 combination cases of thrombotic events associated with the use of one C1 esterase inhibitor product (Cinryze) in patients with hereditary angioedema. Kadoyama et al. [26] evaluated the susceptibility to hypersensitivity reactions to anticancer agents using parameters based on a Bayesian confidence propagation neural network, and the empirical Bayes geometric mean. These indexes of pharmacovigilance provided signals of mild, severe and lethal hypersensitivity reactions associated with paclitaxel and docetaxel agents. In another study [27], the same group demonstrated with the same approach that carboplatin and oxaliplatin caused mild, severe, and lethal hypersensitivity reactions, whereas cisplatin did not. The use of dexamethasone affected oxaliplatin-induced mild hypersensitivity reactions, but had lesser effects on severe and lethal reactions. These findings highlight the significant potential of SC models to analyze huge amounts of data and the ability to discover patterns deeply hidden within the data. Another three studies highlighted the importance of a diagnostic tool for assessment of adverse drug events (BARDI). This computer program is based on BN and is able to perform a differential diagnosis on cutaneous reactions suspected of being drug-induced [28–30].

### Studies on other allergic diseases

Finally, the review revealed that few studies about SC models and other allergic diseases are present in literature. In 2015 Christopher et al. [31]. presented a CDSS based on ANN to assist junior clinicians to diagnoses the presence or absence of allergic rhinitis analyzing reports of intradermal skin tests. The trained neural network achieved an accuracy of 88, 31%. In another work Goulart et al. [32] proposed the ANN to study an allergic conjunctivitis screening questionnaire. In this work the ANN predicted allergic diagnosis in 100% of cases using 7 of the 15 existent items. A study conducted in Japan by Takahashi et al. [33], proposed the use of ANN to predict the effects of atopic dermatitis in infancy from an epidemiological survey. A total of 4610 answered surveys were received: 2714 from mothers of infants (12 months old) and 1896 from mothers of children (2 years old). The sensitivity, specificity and predictive accuracy of the ANN model were respectively 88.6, 99.5 and 96.4%.

## Discussion

### Main findings

This systematic literature review explored the main SC methodologies to investigate allergic diseases. Results were obtained after an exhaustive literature research and examination of hundreds of papers focused on clinical trials. Several studies provided results about SC systems focused on early diagnosis of allergic diseases and the classification of illness categories (i.e., exacerbation, severity) obtaining a mean accuracy of 86.5%. More specifically, this review revealed that the SC approach could have an important impact on the analysis of an enormous amount of screening questionnaires and in the prediction of allergic diseases, discovering patterns deeply hidden within the still-unexplored data [32, 33]. This is possible because the SC models are flexible and able to generalize and predict on an individual basis the probability of diagnosis related to the specific disease of questionnaire respondents. In this specific case, the ANN and BN models are particularly suitable. Another important issue that the use of SC models can resolve concerns missing data in clinical trials. Frequently patients do not complete their follow-up according to a protocol for a variety of reasons, making data analysis more difficult. The BN is a good example of models naturally suitable for handling missing data, as suggested by Carpenter et al. [34, 35]. Another important result emerging from our review is that the SC models could have an important role in CDSS. Indeed, they provide an opportunity to assess the overall information of the main phenomena coming from patients (i.e., identifying information, family history, environmental exposure, perceived treatment efficacy, disease-related quality of life questionnaire) and clinicians (i.e., laboratory tests, values of spirometry), underlying critical features of the disease, treatment planning and the provision of warnings by adding new evidence through associative recall from historical data. In this regard, studies on clinical emergence coupling CDSS-human interaction with the clinician’s knowledge and the suggestions of feedback signals are undertaken in order to generate patient-specific advice and to assist clinicians at the point of care [14, 31, 36]. SC models are also suitable for analyzing data when the likelihood is not defined and statistical tests are not appropriate. More specifically, the choice of an FL approach in substitution of other SC models can provide valuable support, since it starts from the context of a lack of experience data and entangled cause-and-effect relationships, which make it difficult to assess or diagnosis allergic diseases at an early stage [8, 16]. FL is extremely flexible, allowing the decision maker to use a broad range of linguistic variables and modifiers for finer discrimination. It is also a useful system in the case of the presence of a series of sub-decisions where available data is based on vagueness, uncertainty and opinion, such as questionnaires.

### Strengths and limitations

Strengths of this review are related to an exhaustive literature search in PubMed and ScienceDirect databases. The research was performed following PRISMA guidelines and using recommended search queries with consensus finding. Moreover the protocol of this review was registered within the PROSPERO database, with the code CRD42016038894. We found most of SC works deal with asthma, six studies about ADR, and few studies about other allergic diseases. In this regard, in some cases their accuracy although high was quantitatively synthesized on few works. This result reveals how the SC approach is widely used to diagnose asthma, but it is still largely unexplored for other hypersensitivity diseases. Moreover, there are also limitations with the studies themselves. To date, a comparative analysis of SC performances with classic statistical methods was not possible due to the lack of studies comparing these models against a benchmark. This is due to the fact that in most cases the use of these advanced techniques needs to overcome obstacles including the need to establish multidisciplinary teams [37], the resistance to change in working practice especially from older clinicians [38] and the lack of appropriate gold standard clinical assessment procedures [39].

## Conclusions

This systematic review, analyzing clinical trials employing SC methodologies, shows as these methodologies have been used in allergy field for several purposes such as for detecting patients with asthma exacerbations, to prompt clinicians to identify guideline-eligible patients, to evaluate putative ADR, to discriminate drug from nondrug-induced reactions, to improve the diagnostic accuracy and to enhance the management of patients with hypersensitivity reactions. The review also identify promising trends, especially in the diagnosis, prognosis and treatment of some allergic diseases, but also the need for a more extensive application as occurs in genetic association studies [40–43].

Such methods enable a new concept for modeling allergic diseases that combines the collection and mining of multimodal clinical evidence, with dynamic modeling of causal factors. We believe that the introduction of SC models can ease the exploration of big clinical data sets to enable better understanding of allergic disease subgroups, their pathophysiology and optimization of existing treatments. Clinicians should improve evidence by undertaking more randomized controlled trials to prove the efficacy of SC methodologies. In this regard, they should be trained to be more confident with the new perspective provided by these advanced techniques going over their standard methods of choice in interpreting medical data. The review provides evidence that SC methodologies can play a key role in predicting the onset, diagnosing, evaluating pathogenesis and prognosis and managing most of allergic diseases. Moreover, these methods can discover new patterns and evidence about early recognition of inflammatory markers and their relation to allergic diseases [44]. Nowadays most studies deal with asthma, however it is to be hoped that in the near future SC methodologies could be used to investigate all allergic diseases, with a particular attention to those pathologies with a huge burden on health for their impact on quality of life and their severity, such as urticaria and anaphylaxis.
